# Brazilian Immigrant Parents’ Perspectives on Oral Health in Early Childhood and Suggested Strategies for Education, Access, and Care: Qualitative Study

**DOI:** 10.2196/78835

**Published:** 2026-04-07

**Authors:** Ana Cristina Lindsay, Denise Lima Nogueira, Steven A Cohen, Mary L Greaney

**Affiliations:** 1 Department of Urban Public Health, Robert and Donna Manning College of Nursing and Health Sciences, University of Massachusetts Boston, 240 Morrissey Blvd, Boston, MA, 02125-3450, United States, 1 (617) 287-5000; 2 School of Nursing, Faculty Luciano Feijão, Sobral, Brazil; 3 University of Rhode Island, Kingstown, RI, United States

**Keywords:** oral health disparities, oral health promotion, preschool-aged children, Brazilian immigrant families, qualitative methods, culturally tailored interventions, community-based education, access to dental care

## Abstract

**Background:**

Oral health in early childhood is vital for long-term well-being, yet dental caries is highly prevalent among young children in the United States, especially in low-income and immigrant families. Brazilian immigrants, a rapidly growing Latin American population in the United States, face distinct barriers to oral health care, such as language differences, limited access to care, and a lack of culturally tailored resources. Despite this, Brazilian immigrants are understudied in public health research. Understanding Brazilian immigrant parents’ perspectives is essential to advancing oral health equity through culturally responsive strategies.

**Objective:**

This study aims to understand (1) parents’ views on the best ways to support their children’s oral health, (2) perceived barriers to oral health care, and (3) preferred methods and strategies for addressing barriers and receiving oral health education and care.

**Methods:**

This qualitative study usedin-depth, semistructured interviews with Brazilian immigrant parents. Guided by the social ecological model, the data were thematically analyzed to identify multilevel influences on oral health behaviors as well as intervention preferences.

**Results:**

Forty-eight Brazilian immigrant parents (29 mothers and 19 fathers) participated. Most had low acculturation levels and lived in primarily Portuguese-speaking households. Parents stressed the need for early, community-based oral health education in schools and daycares. They preferred visual and digital materials, such as videos, apps, and cartoons in Portuguese to overcome language barriers. Parents spoke of language and cultural challenges, limiting access and reducing confidence in navigating care. Access to affordable dental services was a major concern. Parents supported expanded school-based services and culturally welcoming care. Notably, mothers often focused on home routines and navigating systems, while fathers emphasized community outreach and structural barriers.

**Conclusions:**

Brazilian immigrant parents called for linguistically and culturally tailored oral health programs to be offered in trusted community settings, along with improved dental care access through policy changes and expanded insurance coverage. Multilevel strategies addressing both behavioral and systemic barriers show promise in reducing disparities. Future efforts should prioritize the development and evaluation of scalable, culturally responsive models that meet the needs of Brazilian immigrant families.

## Introduction

Oral health in early childhood is a critical component of overall child well-being, with lifelong implications for physical, emotional, and social development [1-3]. Despite national efforts to reduce oral health disparities, dental caries is the most common chronic disease among young children in the United States, disproportionately affecting low-income and racial or ethnic minority populations, including immigrant families [4]. According to a recent report by the Centers for Disease Prevention and Control, approximately 11% of children aged 2 to 5 years have one or more primary teeth with untreated decay, and this prevalence is higher among Latino children (eg, Mexican Americans, 18%) [4].

Brazilian immigrants are one of the fastest-growing Latin American populations in the United States yet remain largely understudied in public health research [5]. In recent years, the Brazilian immigrant population in the United States has grown significantly. According to the US Census Bureau, the Brazilian population nationwide has nearly tripled over the past 2 decades, making it one of the fastest-growing immigrant groups [5,6]. Massachusetts is home to approximately 130,323 Brazilian residents—the second-largest Brazilian population in the United States, surpassed only by Florida [5].

Despite this growing presence, Brazilian immigrants often face unique challenges that may not always be addressed in broader Latinx immigrant health research [5,7]. One key distinction is linguistic: Brazilian immigrants primarily speak Portuguese, which sets them apart from other Latin American populations who speak Spanish. This language difference creates specific communication challenges in accessing health care services, including oral health care. It also limits the effectiveness of educational materials and interventions designed for Spanish-speaking populations [5,7].

Much of the existing research on immigrant oral health in the United States focuses on Spanish-speaking communities [8-12], and there remains a limited body of research focused specifically on the oral health of Brazilian immigrant families [6]. Emerging evidence suggests that cultural norms, language barriers, limited access to care, and lack of familiarity with the US health care system can present significant challenges to maintaining oral health among children in Brazilian immigrant households [6].

This research gap presents a significant barrier to developing effective, culturally tailored interventions. Given that cultural and linguistic factors play a critical role in shaping health behaviors and outcomes, especially in oral health, addressing this gap is essential for improving health equity among Brazilian immigrants [6,13,14].

Parents play a central role in shaping their children’s oral hygiene behaviors and use of dental services [15-18]. Immigrant parents often face unique systemic and structural barriers that may undermine even strong intentions to support their children’s oral health [19-22]. Moreover, public health messaging and health care delivery systems often fail to accommodate the linguistic and cultural needs of Portuguese-speaking immigrant communities [7]. For Brazilian immigrant families in the United States, the intersection of cultural practices, acculturation stress, language barriers, and service inaccessibility creates distinct oral health challenges that are not addressed by most health education efforts [7,23-25].

Community-based and culturally responsive approaches are essential to advancing oral health equity for immigrant populations [19-22]. Frameworks, such as the social ecological model (SEM), provide a valuable lens for understanding how individual, interpersonal, organizational, community, and policy factors influence oral health behaviors and access to dental care [26]. While much of the existing literature has focused on knowledge deficits or service utilization patterns, fewer studies have explored immigrant parents’ ideas and recommendations for improving oral health, especially through multilevel intervention strategies addressing education, access, and culturally appropriate care [27-30].

To address noted gaps, this study explored the perspectives of Brazilian immigrant parents living in Massachusetts regarding the challenges and opportunities for promoting early childhood oral health. Specifically, we aimed to understand: (1) parents’ views on the best ways to support their children’s oral health, (2) perceived barriers to oral health care, and (3) preferred methods and strategies for addressing barriers and receiving oral health education and care. Grounded in the SEM, this qualitative study provides insights that can inform the design of effective, community-based interventions to promote oral health and health equity among Brazilian immigrant populations.

## Methods

### Study Design

This qualitative study used in-depth, semistructured interviews. In line with qualitative research traditions, this approach enabled the collection of rich, contextualized narratives illuminating how cultural values, parenting practices, and systemic barriers shape health-related behaviors and decision-making in immigrant communities [31,32].

### Participant Eligibility and Recruitment

ligibility criteria included (1) self-identifying as a Brazilian immigrant parent, (2) being at least 21 years old, (3) having at least 1 child aged 2 to 5 years, (4) residing in Massachusetts, and (5) having lived in the United States for at least 6 months to ensure sufficient exposure to local health care and cultural systems. This 6-month minimum was required to help ensure participants had adequate time to engage with the US health care system and broader social environment. We aimed to capture insights from parents who had begun navigating pediatric and dental care, encountered potential access barriers, and could reflect on how US-based experiences compared with those in Brazil. Additionally, only 1 parent per household was eligible to participate, ensuring that each interview represented a distinct family unit.

Participants were recruited using purposive sampling, in partnership with local organizations that conducted community-based outreach by distributing study flyers in Portuguese and posting in Brazilian-focused Facebook and WhatsApp groups [33]. Additionally, participants were recruited using snowball sampling [33]. Interested individuals contacted the research team by text message to confirm eligibility and schedule an interview [33].

### Data Collection

Two native speakers with postgraduate training in public health and maternal and child health and extensive experience in qualitative research and engaging with immigrant communities conducted all interviews in Portuguese. One interviewer is a Brazilian immigrant to the United States and a nonpracticing dentist, while the other is a nurse with strong professional ties to the Brazilian community in Massachusetts. Their cultural fluency and professional backgrounds supported a respectful, informed, and empathetic approach to interviewing, contributing to the credibility and depth of the data collected [34].

Interviews were conducted between December 29, 2023, and March 31, 2024. All interviews were conducted via Zoom, a secure video conferencing platform, to ensure participant flexibility and minimize common logistical barriers, such as transportation and childcare. This approach enabled participants to join from a location of their choice, fostering comfort and convenience while maintaining face-to-face interaction, which can increase rapport-building in qualitative research. Before the start of each interview, the interviewer informed participants in Portuguese of the study’s purpose, procedures, and their rights, including the voluntary nature of participation and their ability to withdraw at any time without penalty. Verbal informed consent was obtained in Portuguese before proceeding with data collection.

Interviews were conducted using a guide developed based on the SEM and previous research on immigrant health [26,35]. The guide was pilot tested for cultural relevance and clarity. The interview guide was designed to explore three domains: (1) parents’ views on the best ways to support their children’s oral health, (2) perceived barriers to oral health care, and (3) preferred methods and strategies for addressing barriers and receiving oral health education and care [36-41].

Before each interview, and after obtaining informed consent, participants completed an interviewer-administered sociodemographic questionnaire. The questionnaire, previously validated and used in studies with Brazilian immigrants in the United States, captured key information including age, marital status, educational attainment, annual household income, number of children aged 2 to 5 years, primary language spoken at home, and length of US residency [7]. The interviewer used Qualtrics to access the survey and enter the data.

To assess acculturation, the survey also included the 12-item Short Acculturation Scale for Hispanics (SASH), adapted for Portuguese-speaking populations [42]. The SASH is a validated tool that measures acculturation through 3 subscales: language use, media preferences, and ethnic social relations [42]. Responses are scored on a 5-point Likert scale, with average scores of 2.99 or higher indicating higher levels of acculturation [42,43]. This scale has demonstrated strong reliability across domains (eg, *α*=.92 overall, .89 for language use, .88 for media preference, and .72 for social relationships) [42,43].

Each interview lasted approximately 40 to 60 minutes and was audio-recorded with the participants’ consent. Field notes were also taken to document nonverbal cues, contextual observations, and interviewer reflections [32]. This supplemental information enriched the analytic process and contributed to the rigor of the qualitative approach. Data collection continued until thematic saturation was reached, when no new insights or themes emerged from subsequent interviews [32].

### Data Analysis

Transcripts were deidentified to ensure confidentiality and checked for accuracy against the original audio recordings by a bilingual member of the research team. A thematic analysis approach was used to identify and interpret patterns within the data [44]. This method was selected for its flexibility and ability to capture both manifest (explicit) and latent (underlying) meanings in participants’ narratives [44,45].

The analytic process began with repeated readings of the transcripts to allow for immersion in the data and develop a holistic understanding of participants’ experiences [32,44,45]. A preliminary codebook was developed using a hybrid approach, which incorporated deductive codes informed by the study’s theoretical framework (SEM) alongside inductive codes that emerged directly from participants’ responses [26,32,44,45]. This approach allowed the team to remain theoretically grounded and open to new or unexpected insights.

Two bilingual researchers independently coded the transcripts line by line using MAXQDA qualitative analysis software (VERBI Software GmbH), which facilitated the application, retrieval, and organization of codes [46]. Coding was conducted in Portuguese to preserve cultural nuance and linguistic fidelity. Following initial coding, the researchers met regularly to compare coding decisions, discuss discrepancies, and refine the codebook through consensus [32,44,45]. Throughout the analysis, the team used a constant comparison method, revisiting previously coded transcripts in light of emerging data to ensure consistency and depth [44,45]. Codes were grouped into broader categories and synthesized into overarching themes that captured the core elements of participants’ perspectives and lived experiences. Analytic memos were written throughout the process to document decision-making, emerging insights, and the evolution of thematic interpretations [44,45]. After identifying the themes, we examined them for differences based on family income, acculturation level (measured by length of stay in the United States), and gender.

To enhance the trustworthiness of the findings, we used several validation strategies [44,45]. These included the use of dual independent coders, regular consensus meetings to resolve discrepancies, and ongoing analytic memo writing to reflect on interpretations and researcher positionality. Although we did not conduct member checking or formal data triangulation, the analysis was further strengthened by the constant comparative approach and by the cultural and linguistic concordance between the researchers and participants, which supported nuanced interpretation and contextual sensitivity.

Selected quotes were translated into English for reporting purposes. To ensure linguistic and cultural accuracy, a back-translation process was used: a second independent bilingual researcher translated the English quotes back into Portuguese, and any discrepancies were reviewed and resolved by consensus. This process helped preserve the original meaning and cultural nuances of participants’ statements.

Sociodemographic data were summarized using descriptive statistics, including means, standard deviations, frequencies, and percentages. These analyses were conducted using SAS version 9.4 [47].

### Ethical Considerations

This study was approved by the University of Massachusetts Boston Institutional Review Board (IRB protocol number 3541, approved June 26, 2023). All participants provided informed verbal consent. The data were stored securely and accessed only by the research team. Participant confidentiality and the right to withdraw without penalty were emphasized throughout the study. Participants received a US $40 gift card in appreciation of their time and contribution to the study.

## Results

### Sociodemographic Characteristics of the Sample

In total, 48 Brazilian immigrant parents (29 mothers and 19 fathers) participated in the study. As seen in Table 1, the participants’ mean age was 36.5 years (SD 6.6 years), with fathers being older on average (39.1, SD 6.8 years) than mothers (33.9, SD 6.5 years). Most (n=28, 58.3%) participants identified as mixed race, followed by White (n=15, 31.3%) and Black (n=5, 10.4%).

**Table 1.** Sample characteristics.

Variables	Total (N=48)	Fathers (n=19)	Mothers (n=29)
Age, mean (SD)	36.5 (6.6)	39.1 (6.8)	33.9 (6.5)
Race, n (%)			
White	15 (31.3)	6 (31.6)	9 (31)
Black	5 (10.4)	1 (5.3)	4 (13.8)
Mixed race (pardo or mestizo)	28 (58.3)	12 (63.1)	16 (55.2)
Marital status, n (%)			
Married or living with partner	41 (85.4)	19 (100)	22 (75.9)
Divorced or separated	3 (6.3)	0 (0)	3 (10.3)
Single	4 (8.3)	0 (0)	4 (13.8)
Educational attainment, n (%)			
Less than high school diploma	10 (20.8)	6 (31.6)	4 (13.8)
High school graduate	23 (47.9)	7 (36.8)	16 (55.2)
More than high school	15 (31.3)	6 (31.6)	9 (31)
Household income per year (US $), n (%)			
<45,000	14 (29.1)	6 (31.6)	8 (27.6)
≥45,000 to <65,000	25 (52.1)	11 (57.9)	14 (48.3)
≥65,000	9 (18.8)	2 (10.5)	7 (24.1)
Number of children between 2 and 5 years old in the household, n (%)			
1	34 (70.8)	9 (47.4)	25 (86.2)
2	14 (29.2)	10 (52.6)	4 (13.8)
Born in Brazil, n (%)			
Yes	48 (100)	19 (100)	29 (100)
States of origin, n (%)			
Minas Gerais	28 (58.2)	8 (42.1)	20 (69)
São Paulo	4 (8.3)	2 (10.5)	2 (6.9)
Espírito Santo	5 (10.5)	2 (10.5)	3 (10.2)
Paraná	2 (4.2)	0 (0)	2 (6.9)
Amazonas	1 (2.1)	0 (0)	1 (3.5)
Rio Grande do Norte	1 (2.1)	0 (0)	1 (3.5)
Bahia	4 (8.3)	4 (21)	0 (0)
Rondônia	1 (2.1)	1 (5.3)	0 (0)
Alagoas	1 (2.1)	1 (5.3)	0 (0)
Rio de Janeiro	1 (2.1)	1 (5.3)	0 (0)
Years of residence in the United States, n (%)			
<5	27 (56.3)	13 (68.5)	14 (48.3)
>5 to <10	16 (33.3)	4 (21)	12 (41.4)
>10	5 (10.4)	2 (10.5)	3 (10.3)
Primary language spoken at home, n (%)			
Portuguese	48 (100)	19 (100)	29 (100)
SASH^a^, n (%)			
Low acculturation (<2.99)	47 (97.9)	19 (100)	28 (96.6)
High acculturation (>2.99)	1 (2.1)	0 (0)	1 (3.4)
Health care insurance, n (%)			
Public or government-sponsored	44 (91.7)	17 (89.5)	27 (93.1)
Private	4 (8.3)	2 (10.5)	2 (6.9)
Dental care insurance, n (%)			
Yes (MassHealth)	33 (68.7)	17 (89.5)	16 (55.2)
No	15 (31.3)	2 (10.5)	13 (44.8)

a SASH: Short Acculturation Scale for Hispanics.

The majority (n=41, 85.4%) of the participants was married or living with a partner. All participants were born in Brazil, with 58.2% (n=28) originating from the state of Minas Gerais. Over half (n=27, 56.3%) had lived in the United States for less than 5 years. Portuguese was the primary language spoken at home, and 97.9% (n=47) scored below 2.99 on the SASH scale, indicating low acculturation to the United States.

Educational attainment varied among participants, with 79.2% (n=38) having completed high school or more. About half reported an annual household income between US $45,000 and US $65,000 (n=25, 52.1%). Most parents (n=34, 70.8%) had one child aged 2 to 5 years, while 29.2% (n=14) had 2 children in that age range. The majority of participants (n=44, 91.7%) was enrolled in public or government-sponsored health insurance programs, and 68.7% (n=33) reported having dental insurance.

### Themes

#### Overview

**Figure 1.**
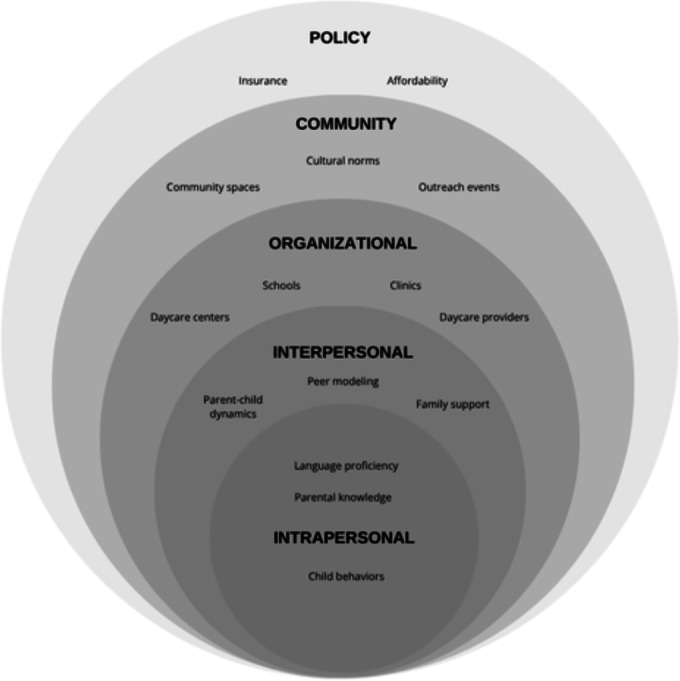
Social ecological model of influences on Brazilian immigrant parents’ perspectives on oral health in early childhood and suggested strategies for education, access, and care.

The analysis revealed a range of factors influencing Brazilian immigrant parents’ perspectives on early childhood oral health. These findings are organized according to the SEM. A conceptual model (Figure 1) visually depicts the SEM levels along with the key themes and subthemes that emerged from the analysis. Representative quotes from parents are integrated throughout the text to illustrate the identified themes and highlight the diversity of perspectives. Notably, gender-based differences were observed in some thematic areas: fathers more frequently emphasized the importance of community outreach and school-based interventions, whereas mothers often focused on home-based education and the challenges associated with navigating health care systems. Differences also emerged by length of time in the United States and income, which further shaped parents’ perspectives and experiences.

#### Intrapersonal Factors: Knowledge Gaps, Language Barriers, and Suggested Solutions for Oral Health

##### Barriers and Challenges to Children’s Oral Health

Parents identified limited knowledge and language obstacles as barriers that they and other Brazilian immigrant families face in supporting their children’s oral health. Several noted that caregivers often lack basic information about pediatric dental care. As one mother explained, “There are many mothers who are not educated on how to take care of their kids’ teeth. They don’t know what’s important*”* (mother #12).

Language barriers were also widely reported, particularly among those newer to the United States. Participants spoke limited English proficiency made it difficult to understand dental recommendations, navigate insurance, or communicate with providers, undermining parents’ confidence and ability to advocate for their children. One mother shared, “I’m not fluent in English. It would help to have information in our language” (mother #7). Another parent stressed the need for organizations that support families struggling with language challenges, “There should be places that help families like ours understand things better, especially when we don’t speak the language well” (mother #14).

Parents who had lived in the United States for fewer than 5 years more frequently reported these difficulties. This highlights how language barriers and cultural unfamiliarity compounded challenges in accessing timely and appropriate dental care.

##### Preferred Strategies to Address Barriers

To address key gaps, parents emphasized the importance of parents and children being provided culturally tailored oral health education especially in Portuguese, when their children are very young to build knowledge and support home-based routines, such as brushing, limiting sugary snacks, and modeling good habits. One mother explained, “Children need encouragement, so that in the future they don’t end up spending money fixing dental problems—if they take care of their teeth consistently” (mother #11).

Parents felt that community-based programs were the most likely to be effective. Parents called for “more educational programs” (mother #19), and one father described the need for initiatives “showing the importance of oral health for children and families, and the damage of not taking care of your mouth and teeth” (father #10). These programs were viewed as particularly important for families new (<5 years) to the United States.

Digital and visual learning tools were widely recommended to improve accessibility and engagement. Suggestions included “videos” (father #6), “podcasts” (father #8), “mobile brushing games” (father #14), and “cartoons for children” (mother #18). Mothers generally expressed a stronger preference for visual and interactive materials that they could use together with their children, reflecting their more active roles in day-to-day child care. In contrast, fathers preferred digital formats they could access on their own during commutes or work breaks.

Language remained a significant barrier, particularly for newer immigrants. Participants with lower English proficiency and shorter US residence were more likely to report difficulties related to language and cultural challenges when accessing dental services. Many stressed the need for materials and support in Portuguese to boost their confidence and ability to care for their children’s oral health. As one father said, “If I had information in Portuguese, I’d feel more confident taking care of my kids’ teeth” (father #9), while another noted, “You trust more when someone speaks your language and understands where you come from” (father #5).

### Interpersonal Level: Parent-Child and Social Relationships

#### Barriers and Challenges to Children’s Oral Health

At the interpersonal level, parents described how their relationships with their children, peers, and broader social networks influence oral health behaviors. Key themes included the role of peer modeling and school-based learning in shaping children’s habits, the challenges parents face in modeling oral health behaviors due to limited knowledge, and the importance of informal social networks, particularly within immigrant communities, for sharing information and accessing support.

Many parents noted that children learn by observing and often mimic what they see at school or from peers. For example, one mother said, “When this is taught at school, kids come home wanting to do it. When it’s only from parents, it doesn’t always work” (mother #20). Similarly, a father shared, “Children like to imitate” (father #12), highlighting the strong influence of peer behavior on home routines.

While parents tried to serve as educators, several noted that a lack of oral health knowledge among other adults undermined their ability to model good habits. One mother explained, “... Because there are many mothers who are not educated on how to take care of their kids’ teeth. They don’t know what’s important” (mother #12).

Parents often shared oral health experiences and information within informal community groups. One mother emphasized the role of peer support: “I usually hear about new things from other moms—like which toothpaste is best or when to take my child to the dentist. We really learn from each other” (mother #13). Another highlighted the need for greater connection among parents, stating, “… programs that bring parents together and help us support each other with our children’s daily oral health care” (mother #6).

Parents who had recently arrived in the United States and those with lower household incomes or without dental insurance more frequently emphasized the need for accessible, no-cost community resources. For these families, support through free cleanings, oral hygiene kits, and multilingual education was seen not just as helpful but essential. These parents also described a greater reliance on informal community networks, such as churches or local nonprofits, to meet health-related needs, further underscoring the importance of community-based resources.

#### Preferred Strategies to Address Barriers

To overcome these interpersonal challenges, parents suggested strengthening culturally grounded, community-based programs that engage both families and peer groups. These programs could reinforce positive oral health behaviors by providing education and support within trusted social networks. As one mother expressed, “Programs that support us, helping with our children’s daily oral health care” (mother #19), underscoring the importance of accessible, community-rooted resources.

Parents felt that offering no-cost or low-cost services, oral hygiene kits, and multilingual educational materials through informal networks, such as churches and community centers, could improve access and engagement, especially for recent immigrants and lower-income families. As a father noted about trusted community venues, *“*Churches already bring families together. Adding health workshops there would be very effective” (father #9).

Parents also valued school-based initiatives, seeing them as a way to promote healthy behaviors that children could bring home, helping to bridge gaps in parental knowledge and role modeling. As one mother reflected, “At my daughter’s daycare, they brush her teeth after lunch. They asked me if I wanted them to do it” (mother #8), highlighting the power of early education settings in shaping habits.

### Organizational Level: Institutions and Services

#### Barriers and Challenges to Children’s Oral Health

At the organizational level, parents highlighted the important role of institutions, such as schools, daycare centers, clinics, and pediatric offices, in shaping children’s oral health. However, parents also identified challenges and missed opportunities. Many parents noted missed opportunities in health care settings where oral health promotion and education could be better integrated. For example, some felt that dental health was overlooked during medical visits. One mother mentioned, “Sometimes when I take my child to the doctor, they only check the general health, and nobody asks about their teeth. It feels like dental health is forgotten” (mother #3). Additionally, unfamiliarity and a lack of culturally welcoming environments made navigating health care systems particularly difficult for immigrant families. One mother expressed, “First is information, for sure, but also being welcomed. That’s very important. One of the hardest things when you arrive here is not knowing where to go for help” (mother #10).

#### Preferred Strategies to Address Barriers

Parents strongly supported embedding oral health promotion within early education settings. They praised daycare programs where children’s teeth were brushed after meals, highlighting schools and daycares as critical sites for developing positive habits. For instance, a mother shared, “At my daughter’s daycare, they brush her teeth after lunch. They asked me if I wanted them to do it” (mother #8). Others suggested distributing toothbrushes and toothpaste in schools alongside instruction on proper use. One mother stated, *“*Distribute brushes and toothpaste, and teach children how to use them at school” (mother #5). The influence of peer learning was also emphasized, with 1 parent noting, “When this is taught at school, kids come home wanting to do it” (mother #20).

Despite this enthusiasm, participants noted a lack of oral health programs in local schools. Some reflected on more robust efforts in their home country of Brazil, such as fluoride days and school visits from dentists. As 1 mother recalled, “In Brazil, we had a fluoride day and dentists visiting schools. Here, there’s nothing like that” (mother #9). Parents reiterated the value of school-based efforts not only for education but also for motivating children through peer influence.

Regarding health care environments, parents recommended integrating oral health education into routine pediatric visits and offering workshops at clinics. One mother said, “Offer workshops at the same place where children are seen by doctors. Teach mothers how to prevent diseases too” (mother #12). Continuity of care and easier access to multiple clinics were also viewed as important, with parents advocating for follow-ups similar to pediatric care and more options for dental services.

Welcoming environments were especially valued. Parents appreciated when providers gave tangible tools, such as toothbrushes and toothpaste, helping children get excited about brushing. One mother reflected, “First is information, for sure, but also being welcomed. That’s very important. One of the hardest things when you arrive here is not knowing where to go for help” (mother #10). Another added, “The dentist gave my son a toothbrush and toothpaste. He came home super excited to brush” (mother 23). Relational and culturally sensitive care settings were seen as essential for building trust and encouraging ongoing engagement.

### Community Level: Local Environments and Cultural Context

#### Barriers and Challenges to Children’s Oral Health

At the community level, parents identified environmental, cultural, and informational barriers that limited their ability to support their children’s oral health. These included a lack of localized services, culturally appropriate education, and outreach in trusted spaces. Recent immigrants particularly noted difficulties finding oral health resources in their language or in places they frequented. One mother recommended, “A program that goes into the communities, offering free help. Not everyone has health care or dental insurance” (mother #15). Another parent emphasized the need for local programs: “A more accessible program so that children can get cleanings for free. Some kids don’t even have MassHealth and can’t afford cleanings” (father #7).

#### Preferred Strategies to Address Barriers

Parents were enthusiastic about community-based outreach efforts that could bring oral health education and services into familiar and trusted environments. Health fairs, free hygiene kits, and toothbrushing demonstrations were widely supported. “Health fairs with free kits and toothbrushing demonstrations would really help” (father #6). “The only thing that could help would be providing us with oral hygiene products” (father #3).

Trusted local institutions, such as churches and community centers, were viewed as effective platforms for education and engagement. A mother explained, “Mothers are usually responsible for health in the family. Community centers could offer this support” (mother #11). A father added, “Churches already bring families together. Adding health workshops there would be very effective” (father #9).

Parents also suggested increasing the visibility of oral health messaging through flyers, posters, and small magazines placed in culturally relevant, high-traffic areas, such as supermarkets and bus stops. “Put up posters in places Brazilians go, like supermarkets or bus stops” (father #5). Another noted, *“*Distribute flyers, posters, small magazines with information about oral health” (father #4).

Particularly for those newer to the United States, culturally and linguistically appropriate messaging and delivery channels were vital. In contrast, parents with longer US residency often preferred institutional sources, such as schools and clinics, for receiving health information, suggesting a need for varied community engagement strategies.

### Policy Level: Structural and Systemic Barriers

#### Barriers and Challenges to Children’s Oral Health

At the policy level, parents highlighted structural barriers, particularly related to insurance coverage, service availability, and systemic navigation challenges. Many families described difficulties using public insurance programs, such as MassHealth, due to bureaucratic complexity and long wait times. One mother expressed, “Sometimes you try to make an appointment, and they say, ‘There are no slots. Your insurance isn’t active. You have to call MassHealth...’” (mother #9). Another stated, *“*I never managed to book a dentist for my son through MassHealth. I think there’s little support for the Brazilian community ...” (mother #12).

These experiences reflect what many parents described as system-level inaccessibility, referring to barriers that stem not from a lack of individual effort or community-based resources but from the way the health care system is designed and operates. These include complex insurance rules, difficulty finding providers who accept public coverage, long appointment waiting times, and limited language support within insurance and health systems. For many families, these challenges made it difficult to navigate oral health care, even the services that were technically available.

#### Preferred Strategies to Address Barriers

Parents proposed policy-level changes such as expanding public dental coverage, increasing provider availability, and simplifying the process of booking appointments. For example, one mother suggested, “They should provide more assistance to families, maybe even schedule appointments through the schools” (mother #24), showing support for integrated service delivery.

There were also calls for systemic reforms to improve support for immigrant communities, such as enhanced language access and outreach by state programs. Broader investments in affordable dental services, policy incentives for providers to accept public insurance, and improved transparency around insurance benefits were also suggested to reduce disparities and ensure equitable care. One father stated: “It’s hard for families like mine to get good dental care. Sometimes we don’t undertsand the forms or what the insurance covers. I wish there were programs in our language and more clinics that take public insurance (MassHealth), so everyone could get the care they need” (father #4).

## Discussion

### Principal Results

This qualitative study examined Brazilian immigrant parents’ perspectives on promoting early childhood oral health in the United States, revealing multilevel influences that shape knowledge, skills, attitudes, and behaviors. To our knowledge, based on available literature, this is the first study focusing specifically on this population. Key themes emerged across all 5 levels of the SEM, providing a comprehensive framework to understand how individual behaviors are embedded within broader social and structural contexts. This alignment underscores the need for tailored, multilevel interventions to advance oral health equity among Brazilian immigrant families.

At the intrapersonal level, participants emphasized the critical role of their knowledge and skills in shaping oral health behaviors, highlighting the importance of culturally and linguistically tailored education delivered in Portuguese. Consistent with prior research, parents viewed early education for both children and caregivers as foundational for establishing positive oral hygiene habits [30,48-51]. Digital tools, such as videos, apps, and podcasts, were recommended to complement traditional education and accommodate diverse learning preferences and schedules.

Language proficiency emerged as a key intrapersonal and interpersonal factor influencing parents’ confidence and self-efficacy in navigating oral health care. Limited English skills hindered comprehension and agency, especially among newer immigrants and those with low acculturation, as previous research demonstrates, underscoring the need for linguistically accessible materials and communication [8-12,22].

At the interpersonal level, parents reflected on family dynamics and peer influence. They acknowledged their critical role as educators and role models for their children’s oral health behaviors but also reported knowledge gaps that could limit their effectiveness. Consistent with prior studies, parents noted that children’s peer interactions and school-based learning reinforced positive habits, illustrating how family and social relationships work together to shape behaviors [52-55].

Informal social networks within the Brazilian immigrant community also served as valuable sources of support and information exchange for parents, particularly those recently arrived and with limited access to formal resources. Gendered caregiving roles influenced interpersonal interactions, with mothers often assuming primary responsibility for navigating health care, while fathers focused more on structural access issues. Mothers described needing providers who were patient and approachable due to their caregiving role, highlighting how interpersonal dynamics shape emotional components of provider-patient interactions.

Parents identified schools, daycare centers, clinics, and pediatric offices as key organizational settings for oral health promotion. They advocated integrating oral health education and preventive services into early childhood education environments, emphasizing these institutions’ unique potential to consistently reach children and families. These findings are supported by prior research [52,53,56-59]. Clinics and pediatric providers were viewed as being underutilized and where relational, culturally sensitive care and consistent follow-up could enhance engagement and outcomes.

Participants emphasized the importance of culturally concordant care and bilingual staff, a finding that aligns with previous research highlighting how such practices strengthen patient-provider relationships and improve communication in institutional settings [60-66]. Organizational efforts to recruit Portuguese-speaking staff and create welcoming environments were seen as essential for overcoming barriers related to trust.

At the community level, participants identified trusted venues, such as churches, community centers, ethnic markets, and local events, as vital platforms for oral health outreach. Consistent with prior research, these settings leverage social capital and cultural resonance, fostering engagement and trust beyond formal health care systems [56-59,67-71]. Parents emphasized the importance of providing free or low-cost services and hygiene products within these community spaces, particularly for lower-income families and those without insurance. Participants also suggested combining in-person community initiatives with culturally tailored digital outreach embedded in familiar networks (eg, WhatsApp groups, church social media pages), enhancing accessibility and sustaining engagement across multiple community touchpoints.

At the policy level, systemic challenges, including insurance complexities, cost barriers, and provider shortages, were viewed as major impediments to timely oral health care. Prior research shows that these structural issues disproportionately affect low-income families and recent immigrants, compounding individual and interpersonal challenges [19,21,22]. Parents called for expanded public dental coverage (eg, MassHealth), policy reforms to increase provider availability, and funding for community-based preventive services, such as oral health kits and mobile clinics. The need for bilingual, culturally competent providers and office staff and navigation assistance reflects policy imperatives to address disparities holistically [28,29,36,72].

### Implications for Practice and Policy

The findings emphasize the necessity of coordinated multilevel strategies to effectively promote oral health among Brazilian immigrant families. Practical recommendations include the following:

- Intrapersonal: Deliver linguistically accessible, culturally tailored oral health education and digital resources to improve knowledge and self-efficacy, especially among recent immigrants and those with limited English proficiency
- Interpersonal: Support parental modeling and strengthen peer and family networks as channels for health promotion, while considering gendered caregiving roles and the different relational expectations of mothers and fathers
- Organizational: Integrate oral health into schools, daycare, and pediatric care settings, ensuring culturally competent providers and bilingual staff are available to foster trust and continuity of care. Utilize translation technologies where appropriate
- Community: Leverage trusted community spaces and organizations for outreach, offering free or low-cost services and distributing culturally relevant materials with attention to accessibility for low-income and uninsured families
- Policy: Advocate for expanded dental insurance coverage, increased provider availability, funding for culturally tailored preventive programs, and system navigation supports, such as bilingual oral health navigators and onboarding workshops to assist recent immigrant families.

Special attention is warranted for recent immigrants and economically vulnerable families, which face compounded barriers across all SEM levels. Tailored outreach, considering gendered caregiving roles and flexible service delivery options, such as evening or weekend hours and bundled visits, can improve accessibility and engagement.

Building on these findings, future research should prioritize developing, implementing, and evaluating culturally and linguistically tailored oral health interventions specifically designed for Brazilian immigrant families. Studies could use mixed methods approaches to assess intervention efficacy on behavioral and clinical oral health outcomes over time. Additionally, exploring the role of technology-based solutions, such as mobile health apps and tele-dentistry, may offer scalable ways to overcome language and access barriers.

Further research is also needed to examine intragroup differences by factors such as gender, acculturation level, immigration status, and socioeconomic position to better tailor interventions and policies. Longitudinal studies tracking families’ oral health trajectories as they acculturate could provide valuable insights into how social determinants evolve and impact health outcomes.

At the policy level, advocacy efforts should focus on systematic evaluations of insurance programs and community dental services to identify gaps and inform equitable resource allocation. Implementation science frameworks could guide the translation of culturally competent practices into routine care within community health centers and pediatric clinics serving diverse immigrant populations.

### Limitations

While this study provides rich qualitative insights, several limitations should be acknowledged. The sample consisted of Brazilian parents residing in a specific US state, which may limit generalizability to other geographic areas or immigrant communities [32]. Additionally, the perspectives of those facing the most significant barriers (eg, undocumented parents) may be underrepresented [33,36,72]. Most participants had dental insurance, which might not reflect the experiences of uninsured families. Despite these limitations, this study offers unique and novel insights into the oral health perspectives of Brazilian immigrant parents, a population that has been understudied in this context. Future research could expand to include more diverse immigrant populations and investigate the implementation and effectiveness of the interventions proposed here.

### Conclusions

Brazilian immigrant parents in this study expressed a strong need for linguistically and culturally tailored strategies to support early childhood oral health. Interventions that combine education, improved access to care, and delivery through trusted community settings (eg, daycares, preschools, schools, and churches) offer a promising path to reducing disparities. Parents also emphasized the value of digital platforms, including social media, messaging apps, and online content, as accessible tools for reinforcing oral health messages. Integrating these digital approaches with in-person outreach can improve both reach and cultural relevance. Newly arrived families and those with low acculturation may require additional support in navigating the US dental system, underscoring the importance of onboarding programs and culturally concordant patient navigators. Mothers, often the primary caregivers, face unique logistical and emotional challenges that should inform the design of flexible, family-centered interventions. Policy efforts should focus on expanding access to bilingual, culturally competent providers and investing in both digital and community-based infrastructure to eliminate persistent structural barriers.

## Abbreviations

SASH: Short Acculturation Scale for Hispanics
SEM: Social Ecological Model
